# The Influence of Parents' Background and Their Perception on the Progression of Myopia in Children

**DOI:** 10.1155/2022/4123470

**Published:** 2022-11-24

**Authors:** Zheng-Yang Tao, Shui-Qiang Chen, Yu Tang, Jun Zhao, Jiao Wang, Zhi-Hong Lin, Hong-Wei Deng

**Affiliations:** ^1^Shenzhen Eye Hospital, Jinan University, Shenzhen Eye Institute, Guangdong, Shenzhen, 518000, China; ^2^Shenzhen Aier Eye Hospital Affiliated to Jinan University, Guangdong, Shenzhen, 518000, China; ^3^The Second Clinical Medical College, Jinan University (Shenzhen Eye Hospital), Guangdong, Shenzhen 518000, China; ^4^Shenzhen People's Hospital, The Second Clinical Medical College, Jinan University, The First Affiliated Hospital, Southern University of Science and Technology, Guangdong, Shenzhen, 518109, China; ^5^Affiliated Shenzhen Maternity and Child Healthcare Hospital, Southern Medical University, Guangdong, Shenzhen, 518000, China

## Abstract

**Purpose:**

To study the influence of parents' educational backgrounds and understanding on the progress of myopia in their offspring.

**Methods:**

Spherical equivalent refraction (SE) of the children (aged 6–14) in China was assessed with cycloplegic autorefraction in a two-year longitudinal study. The parents' background information and myopia-related cognition were collected by questionnaires.

**Results:**

The offspring of parents with lower education and more myopic SE had higher myopic progression (mean = –1.42 ± 1.06) than the children of other groups (*P* < 0.05). The parents' understanding of the proper outdoor activity time, sleep duration, reading distance, and indoor illumination for children was not significantly correlated with the progression of myopia in their offspring. The parent's preference for eye care visit frequency had a significant correlation with the myopia development of their children (*r* = 0.076, *P*=0.001^*∗*^). The mean SE progression was −0.84 ± 1.37 and −0.58 ± 1.29 in the children whose parents considered that extracurricular classes would negatively affect myopia development progression and the children whose parents believed it would not, respectively (*P*=0.026^*∗*^).

**Conclusions:**

Most parents misunderstand the influence of insufficient outdoor sports time and extracurricular classes, which require extra near-vision work. Besides, for parents with low educational background and more myopic SE, their offspring had higher myopia progression and may be the key group for myopia control. Finally, parents may obtain life advice and knowledge related to preventing myopia after their children become myopic. It may be of positive significance if this process could take place before myopia onset.

## 1. Introduction

A report from the Brien Holden Vision Institute predicted that by 2050, there would be 4,758 million people with myopia (49.8% of the world's population) [1]. In 2013, the World Health Organization (WHO) highlighted the prevention of myopia in the “Global Initiative for the Elimination of Avoidable Blindness” (Vision 2020). The prevalence of myopia in Chinese primary school students was 76%, while in secondary school students, it was over 80% in 2019, according to a recent cross-sectionalschool-based study in Jiangsu Province [2]. In addition, data from the School of Public Health and Institute of Child and Adolescent Health showed that the peak age of myopic prevalence in Chinese children and adolescents was 13 years in 2005 and 11 years in 2014 and has continued to decrease [3]. Controlling the prevalence of myopia among children and adolescents in East Asia, especially in China, is very difficult.

The influence of the environment on myopia onset is not negligible. Children's outdoor activity time is one of the key environmental factors affecting the progression of myopia [4, 5]. A cross-sectional study by the Asian Eye Epidemiology Consortium showed that less outdoor time was associated with myopia (OR = 0.82, 95% CI: 0.75–0.88) [6]. In addition, near-vision work time and distance also greatly affect the prevalence of myopia among children [6–8]. Recently, indoor illumination has received more attention as an important environmental factor in myopia development because of the increasing indoor activity of children, such as heavier schoolwork burdens and much more electronic screen use since the 21st century [9]. In the study by Wen et al., using a wearable device that records the eye-level illumination, higher light intensity during near-vision work was indicated as an independent protective factor in myopic prevalence [8]. Besides, sleep, as an activity that takes up to one-third to one-half of the day, may be an important factor in myopic progression, though it is still controversial [10–13].

These environmental factors affecting the progression of myopia, however, are not entirely uncontrollable. Is there a correlation between parents' awareness of these factors and myopic progression? To our knowledge, this is the first study to explore the relationship between parents' myopia awareness and myopia development in children. In fact, the popularization of knowledge about myopia prevention consumes many social resources, especially in East Asia, where myopia is highly prevalent. For parents with different educational backgrounds and refraction, their offspring may have various progressions of myopia, which may provide critical intervention for precise myopia control. As a qualitative study, it is hoped that the article could help better understand the relationship between parent perceptions and myopia onset in children.

## 2. Methods

### 2.1. Study Participants

In this study, we randomly selected five primary schools in Futian District, Shenzhen, and conducted refraction and visual acuity examinations 3 times on 3,695 students in grades 1–4 from May 2019 to June 2021. After the first examination (including slit lamp and visual acuity examinations), the students who met the inclusion criteria were determined and recorded. The electronic questionnaires related to the common knowledge of myopia were sent to their parents through a specified online platform three days after the last examination was finished.

### 2.2. Exclusion Criteria

Subjects were excluded for the following reasons:

① Poor compliance for the refraction and visual acuity tests
② Loss to follow-up or incomplete data
③ Ocular position abnormality (including strabismus and nystagmus)
④ Abnormality in color perception
⑤ Amblyopia
⑥ Fundus or corneal disease

If the subjects do not meet any of the previously mentioned criteria, they will be included in this study.

### 2.3. Study Design

The electronic questionnaire survey included five questions for collecting parents' background information and six questions about parents' perception of environmental factors that may affect myopia development.

The parents' background information included gender, educational background, age, history of myopia, and the current refraction. In this paper, the following items are included in the questionnaire as the collection of parents' perceptions of environmental factors: the daily outdoor activity time [5, 14–16], the indoor ambient light [9, 17, 18], sleep duration [10, 11], the proper distance of near-vision work, and extracurricular classes (near-vision work burden in addition to schoolwork) [8, 19]. The preference for the follow-up period of the children's eye care was also included in the questionnaire.

### 2.4. Study Procedure

In the first ophthalmic screening, four ophthalmologists and ten optometrists carried out slit lamp examinations (Topcon SL-2G Slit Lamp, Japan), color tests (Kechang Wang, Color Vision Test, People's Medical Publishing House, China), and visual acuity examinations (E chart, WB-1112E, WenBang Co., China) for all students in the indoor gyms or available empty classrooms of the five schools. During this process, any of the subjects who met the exclusion criteria were recorded. The final inclusion list was determined five days after the first screening.

The standard logarithmic tumbling *E* chart was set at 5 meters. After the visual acuity examination, cycloplegia would be induced in both eyes by administering one drop of tropicamide phenylephrine (Santen Pharmaceutical Co., Japan) 10 minutes apart (three drops in total) to obtain adequate mydriasis with the disappearance of the pupillary light response. The sphere diopter, cylinder diopter, and axis were measured by using an auto-refractor (Beijing Dakang Instrument Co., China) and recorded after the measurement. Visual acuity and refraction measurements were performed once a year and followed up for two years.

Electronic links to questionnaires were distributed to parents after the last measurement got finished in June 2021. Parents answered all the questions in the questionnaire via mobile phones within five days. The electronic platform manager then transmitted the data to two quality control personnel. The results of the questionnaire were checked, and the reasons for those who did not complete it were recorded.

### 2.5. Data Analysis

In this study, spherical equivalent refraction (SE) is used to represent the refraction state. Myopia is defined as a condition in which an eye's SE is ≦−0.50 diopters sphere (DS) when ocular accommodation is relaxed [20]. The statistical package R software for Windows (version: 3.5.2, Ross Ihaka and Robert Gentleman, University of Auckland, New Zealand) and SPSS statistical software (version: 25.0, IBM Co., New York, USA) were used for analysis.

## 3. Results

This study included 3,695 Chinese primary school students from May 2019 to June 2021. A total of 1502 individuals were excluded for lost to follow-up (951 individuals) or incomplete questionnaire data (551 individuals). “Incomplete questionnaire data” means that the parents did not fill in all the questions in the questionnaire, of which “parent's refraction” was the most unanswered question. This may be related to the fact that these parents have never had an optometry test. “Lost to follow-up” referred to the students who did not finished all the refraction measurements during the two years of follow-up for personal reasons (439 individuals), such as physical problems, changing schools, dropping out, and some other reasons or for the epidemic prevention policies during COVID-19 (512 individuals). Fifteen individuals were excluded for poor compliance for the diopter and visual acuity tests, and a total of 138 individuals were excluded for the abnormal color perception, 144 were excluded for ocular position abnormalities (strabismus: 123, nystagmus: 21), 81 were excluded for amblyopia, and 25 for fundus disease. Finally, 1,790 children (3,580 eyes) were available for the final analysis.

At the baseline, 837 (46.7%) right eyes and 734 (41.0%) left eyes were defined as myopic. There was a high correlation between right and left eye refraction (Spearman's rank correlation coefficient: baseline, *n* = 1789, *r* = 0.789, *P* < 0.001; 1 year, *n* = 3620, *r* = 0.825, *P* < 0.001; 2 years, *n* = 3694, *r* = 0.818, *P* < 0.001); therefore, only the results from the right eyes are shown. There is a significant correlation between children's age and SE, so age would be considered an important correction factor in the analysis (Age and SE: *r* = −0.309, *P* < 0.001^*∗*^; Age and SE progression: *r* = -0.049, *P*=0.037^*∗*^). The mean SE was −0.67 ± 1.35 at the baseline, −0.70 ± 1.39 in 2020, and −0.90 ± 1.51 in 2021. The other demographic information for this study is shown in Table 1.

The subjects without myopia at the baseline (*n* = 840) were divided into two groups based on whether one of the two eyes developed myopia (denoted as the “DM group,” *n* = 431) or not (denoted as the “NDM group,” *n* = 409) during the two years of follow-up.

### 3.1. Part 1. The Parents' Understanding of Myopia Shown in the Questionnaire

**Figure 1:**
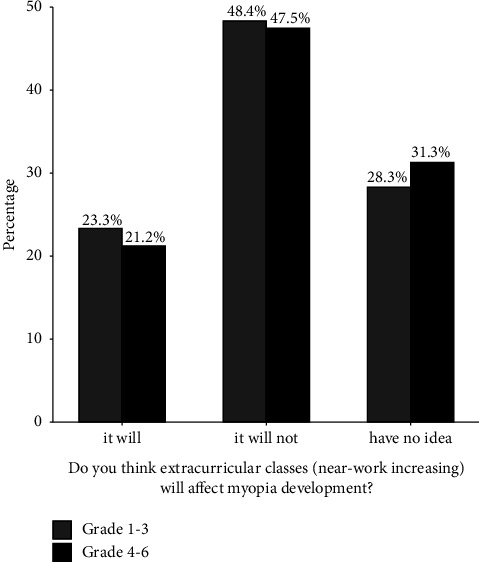
The parents' attitude towards near-vision work required in extracurricular classes.

In the answers about outdoor exercise time, 47.37% (*n* = 848) of parents thought the time should be more than two hours, 40.79% (*n* = 730) thought the time should not be less than one hour, and only 11.84% (*n* = 212) thought less than one hour of outdoor activity is enough for children. As for the proper distance of near-vision work, 72.77% (*n* = 1,303) of parents knew that their children should keep a reading distance of at least 33 cm, 18.27% (*n* = 327) thought it was acceptable when their children's reading distance was less than 33 cm, and 8.96% (*n* = 160) did not pay attention to the proper reading distance. However, 22.52% (*n* = 403) of parents regarded extracurricular classes that require significantly increased near-vision work time (such as musical instrument classes, painting classes, and so on) as a negative factor in myopia development, 48.01% (*n* = 859) thought that it would not have any adverse effect on children's myopia, and 29.47% (*n* = 528) answered “unclear.” Figure 1 shows the parents' attitudes (in percentage) toward extracurricular classes when the answers were divided into two groups based on students' grades.

As for indoor illumination, most of the parents (90.78%, *n* = 1,625) thought that reading illumination was one of the important factors affecting myopia, while 1.24% (*n* = 22) and 7.98% (*n* = 143) thought that it was not or probably not a correlated factor. Next, 84.95% (*n* = 1,521) of parents thought that having their children get 8–10 hours of sleep was beneficial for preventing myopia onset, 8.17% (*n* = 146) thought that sleep duration should be more than 10 hours, and 6.87% (*n* = 123) believed that less than eight hours of sleep would not be harmful to their children's myopia development.

**Figure 2:**
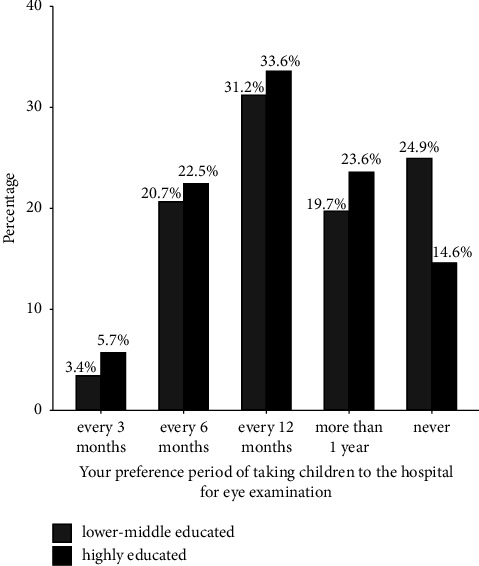
The parents' preference period of eye care visits for their children.

Last, there is a relatively large preference difference in the frequency of parents taking their children to the hospital for an eye examination. 5.14% (*n* = 92) of parents preferred to bring their child for a routine eye exam every three months, while 21.90% (*n* = 392) preferred six months, and 32.96% (*n* = 590) preferred 12 months. Around 22.63% (*n* = 405) of parents thought that taking their children to a routine eye exam for 12 months or more would not be a big problem for myopia development, and 17.37% (*n* = 311) never took their children for an eye exam.  Figure 2 shows the parents' attitudes toward eye examination (in percentage) when the answers were divided into two groups based on the parents' educational background.

### 3.2. Part 2. The Correlation between Parents' Background Information and Children's Myopia Situation

In our data, children's myopia progression in the group of parents without a history of lens prescription (mean value = −0.41 ± 1.12) was significantly slower than in the group of parents having a spectacle-wearing history (mean value = −0.70 ± 1.23, by the Wilcoxon rank sum test, *P* < 0.001^*∗*^). This was consistent with previous studies [21, 22]. By using a partial correlation coefficient for analysis, parents' SE showed a significant correlation with the progression of myopia in children after correcting age and children's baseline SE factor (*r* = −0.135, *P* < 0.001^*∗*^). Parents with a bachelor's degree made up the highest proportion among those who completed the questionnaire. However, the Wilcoxon rank sum test indicated no significant difference in children's myopic progression with the parents' educational backgrounds. The previous results of children's myopic progression changing with the parents' background information are shown in Table 2. Besides, correlation coefficient analysis showed that the educational background of parents has a significant correlation with the SE value of the parents themselves (*r* = 0.135, *P* < 0.001), while there was no significant correlation between the different educational backgrounds of parents and children's myopic progression after correcting age, children's baseline SE, and the parent's SE factor (*r* = –0.006, *P*=0.087).

The parents were further divided into groups according to their SE values and educational backgrounds. The SE values greater than or equal to −3.0 DS were classified as a nonlow myopia group, and the rest were classified as medium-high myopia. In addition, those with a bachelor's degree or higher were classified as highly educated, while the rest were classified as the lower-middle educated group. As shown in Table 3, the Kruskal–Wallis rank sum test indicated that the two-year myopia progression of the offspring had a significant statistical difference among the four groups, and the greatest difference was found in the lower-middle educated and medium-high myopia groups (mean = −1.42 ± 1.06), which showed a significant statistical difference compared with the other three groups (by the chi-square test).

### 3.3. Part 3. The Relationship between Parents' Awareness of Myopia and Children's Myopic Situation

Spearman's rank correlation test was applied to analyze the correlations between myopic progression and the parents' answers, which could be transformed into hierarchical data, as shown in the following paragraph. After correcting age, children's baseline SE, and the parent's SE factor, the parents' preference of their children's outdoor activity time did not show a statistically significant correlation with their myopia development in the two-year follow-up (*r* = −0.029, *P*=0.365), nor the preference of sleep duration (*r* = −0.043, *P*=0.185). However, besides, the preference of eye care duration showed a statistically significant correlation with the myopia development of their children (*r* = 0.076, *P*=0.001^*∗*^).

The Kruskal–Wallis rank sum test was applied to analyze whether there were differences in myopic progression between the parents' attitudes, as shown in their answers in the following paragraph. The mean value of myopic progression was −0.31 ± 0.98 (*n* = 29) in the group of children whose parents believed that the illumination environment during reading would not affect myopia, while the mean value was −0.56 ± 1.19 (*n* = 1,761) for the counterpart. The two attitudes toward the illumination condition of reading did not show statistical differences between the myopic progression of their children (*P*=0.365). Also, for those parents who knew the proper reading distance and those who did not, the mean values of their children's myopic progression were −0.55 ± 1.17 (*n* = 1,605) and −0.70 ± 1.25 (*n* = 185), respectively (*P*=0.226). However, for those parents who considered that extracurricular classes would negatively affect myopia development and those who believed that it would not, the mean values of their children's myopic progression were −0.84 ± 1.37 (*n* = 403) and −0.58 ± 1.29 (*n* = 859), respectively (*P*=0.026^*∗*^).

The Wilcoxon rank sum test with continuity correction was applied to analyze whether there were differences in the parents' attitudes between the DM and NDM groups. In terms of parents' attitudes towards outdoor activities, assign 1 to “children should have outdoor activities less than 1 hour,” 2 to “1-2 hours,” and 3 to “more than 2 hours,” which shows a statistically significant difference between the DM group (mean value = 2.25 ± 0.72) and the NDM group (mean value = 2.36 ± 0.69) (*P* = 0.039). In terms of extracurricular classes, we assign 1 to “it will affect children's myopia development,” 2 to “it will not,” and 3 to “have no idea,” which shows a statistically significant difference between the DM group (mean value = 2.05 ± 0.90) and the NDM group (mean value = 2.16 ± 0.88) (*P* = 0.08). In terms of the eye examination period, we assign 1 to “every 3 months,” 2 to “every 6 months,” 3 to “every 12 months,” 4 to “every 1 year,” and 5 to “never,” which shows a statistically significant difference between the DM group (mean value = 3.22 ± 1.19) and the NDM group (mean value = 3.43 ± 1.07) (*P* = 0.007). However, in terms of parents' attitudes towards indoor illumination, reading distance, and sleep duration, there were no statistical differences between the DM and NDM groups (*P* > 0.05).

## 4. Discussion

Based on current research, most parents in this study have a relatively correct understanding of indoor illumination, reading distance, and sleep duration [9–11, 17, 18]. However, even after two years of feedback on their child's vision and SE value, about half of the parents still did not realize that two hours of outdoor exercise was necessary to slow the progression of myopia. This may be related to the overall social atmosphere of excessively emphasizing basic education. In China, education from primary school to middle school is compulsory. In most cases, there is no examination required, except for a few key middle schools that still reserve entrance examinations to obtain high-quality students. However, to provide their children with better educational resources, many parents still invest their children's spare time to improve their academic ability rather than outdoor sports.

In addition, with the Chinese restriction policy on after-school classes in recent years, after-school interest-oriented classes, such as music and painting classes that require extra eye use, have become increasingly popular in many developed cities. These classes are deceptive for the increased near-vision stress because parents often believe that only excessive reading tasks (especially schoolwork or homework) can cause myopia. Even if teenagers in Western countries are also keen on acquiring artistic skills, Chinese students' culture courses take up a more significant proportion of school time, rather than physical education lessons and finish later (often around 4-5 pm). Therefore, these additional courses gradually become an integral part of near-vision work and affect myopia prevalence in primary school students. However, in our questionnaire, nearly half of the parents did not think that these classes would have a negative impact on myopia, which did not differ significantly between the children in grades 1 to 3, where they were less stressed, and the children in grades 4 to 6, where they were more stressed (Figure 1), and this thinking may be a mainstream trend in the next few years, especially in some economically developed cities.

The parent's SE value is the primary influence on that of their child. Even though there was some correlation between the parents' SE value and their educational background, it did not show a statistically significant correlation with the SE value of their child. This is consistent with the study of Jan et al. [23]. The children of parents with low education and more myopic SE had higher myopia progression, but there was no difference between this group and the rest of the population in their willingness for eye care follow-up. This may result in the offspring of this group facing a less desirable refractive error situation, leading to a higher risk of developing fundus disease in adulthood. Active early intervention in the offspring of this group may be one of the critical measures in reducing the progress of myopia in the primary school population.

At the beginning of the study design, though the shorter period of routine examination, such as 3 months, does not mean that it is more beneficial to most patients, the frequency to a certain extent can reflect parents' concern for the development of myopia in children. However, in our data, with the preference of the routine eye examination period becoming longer, the SE value of the children tends to be less myopic. Besides, the parents who think that extracurricular interest classes have a negative impact on their children's diopter have greater myopic progression than their counterparts. The final result is worth paying attention to because it does not fit our conventional wisdom. In fact, this result is probably related to the fact that the questionnaire was conducted after the last follow-up of the study. One possible explanation is that the high frequency of eye care visits was caused by the fast SE progression and rapid vision loss in part of the children, rather than spontaneous parental concern about their child's myopic situation. Similarly, parents may have received advice from their eye care provider to reduce indoor extra after-school classes requiring close eye use and other activities that might bring varying degrees of myopic progression. This was also demonstrated in the DM and NDM groups.

There were some limitations to our research. First, the questionnaire was performed after the last follow-up, which was a major deficiency in the experimental design. Parents' perceptions of and behaviors about myopia may change in two years, especially for parents whose children developed myopia during the follow-up period. Therefore, it is difficult to prove the causal relationship between parents' myopia cognition and children's myopia development. Second, in the analysis of the possible risk of a shorter sleep duration to faster myopic progression, our study did not include the time duration to fall asleep, the time duration to get up, and the time duration of different activities during wake-up time into the questionnaire. This is one of the deficiencies of this study, which makes the conclusion of the relationship between sleep duration and myopia progression less convincing. Third, the different attitudes toward the reading illumination condition and reading distance did not show statistical differences in the myopic progression of their children. The results did not change significantly when the subjects were divided into different age or grade groups. This may be caused by the differences between the parents' cognitive level and their actual actions to varying degrees, by which it is difficult to reflect the actual condition of children's growth from the questionnaire in this study and the extent to which these conditions would affect their myopic progression. Thus, future research in this field should include more investigation on the actual measures that parents take for preventing myopic onset in their child to observe the matching degree between the parents' cognition and their actual actions. Besides, the introduction of some objective indicators may be necessary. Last, given the proportion of negative responses to the question, the overall sample may not be sufficient. Due to the need to maintain the continuity of repeated diopter measurements, as well as the consideration of funds, schools, and other factors, the sample size was not further expanded later in this study, which will be improved in further research to increase the convincing of the results.

At present, to reduce the incidence of myopia among children, the primary measures in the field of public health are reducing indoor curriculum, increasing outdoor sports time, carrying out myopia screening examinations, and popularizing myopia-related knowledge. Whether parents have the correct knowledge about myopia can help delay the progression in their offspring is still lacking in big data prospective studies to provide high-quality evidence.

## 5. Conclusion

Most parents have a relatively correct understanding of appropriate indoor illumination, reading distance, and sleeping time based on current research studies. However, most parents still misunderstand the negative influence of insufficient outdoor sports time and extracurricular classes. In addition, for parents with low educational backgrounds and more myopic SE, their offspring had higher myopic progression and may be the key group for slowing down its prevalence. Finally, parents may obtain life advice and knowledge related to preventing myopia after their children become myopic. It may be of great significance if this process takes place before myopia onset.

## Figures and Tables

**Table 1:** Demographic information for this study.

Items	Information
Region	Shenzhen, China
Age composition of children	6–14 y
Included subjects for final analysis	1790 (*n*)
Gender composition of children (male/female)	980/810 (*n*)
Gender composition of children (male/female)	392/1398 (*n*)
Parents myopia situation (Myopia/Nonmyopia)	916/874 (*n*)
Spherical equivalent of children at the baseline	mean = −0.67 ± 1.35 D

*n*: number.

**Table 2:** Children's myopia progression changing with the parents' background information.

Items	Subitems	*N*	Mean ± SD	*P* value
Lens prescription history	Yes	901	−0.70 ± 1.23	<0.001
	No	889	−0.41 ± 1.12	

Parents' SE	>−0.50DS	246	−0.20 ± 1.09	<0.001
	−0.50DS∼−3.00DS	786	−0.63 ± 1.16	
	−3.25DS∼−6.00DS	591	−0.74 ± 1.25	
	<−6.00DS	167	−1.01 ± 1.27	

Educational background	Primary/Junior high school	224	−0.42 ± 1.46	0.102
	High school	308	−0.48 ± 1.09	
	Bachelor's degree	1087	−0.62 ± 1.15	
	Master's degree	171	−0.47 ± 1.12	

*N*: number. Mean ± SD: the mean value ± standard deviation of the children's myopia progression during the two years of follow-up. *P* value: there was statistical significance when *P* value was less than 0.05.

**Table 3:** The differences of myopia progression during the two years of follow-up between parents with different educational backgrounds and spherical equivalent values.

Groups	*N*	Mean ± SD	*P* value (K)	*P* value (vs. LE+ MHM)
Lower-middle educated and nonlow myopia	148	−0.86 ± 1.23	<0.001	0.046
Lower-middle educated and medium-high myopia	150	−1.42 ± 1.06		—
Highly educated and nonlow myopia	810	−0.51 ± 1.14		0.006
Highly educated and medium-high myopia	681	−0.76 ± 1.27		0.033

LE+ MHM: lower-middle educated and medium-high myopia group. *N*: number. Mean ± SD: the mean value ± standard deviation of the children's myopia progression during the two years of follow-up. *P* value (K): the *P* value of comparing the difference of myopia progression among the four groups by the Kruskal–Wallis rank sum test. *P* value (vs. LE+ MHM): the *P* value of comparing the myopia progression of the LE+ MHM group to the other three groups by the chi-square test. *P* value: there was statistical significance when *P* value was less than 0.05.

## Data Availability

The transcripts from which this manuscript was developed are available on request from the corresponding author.
